# Impact of lower-body strength and power on lunge velocity in elite fencers: a comparative weapon analysis

**DOI:** 10.7717/peerj.20879

**Published:** 2026-02-27

**Authors:** Gangrui Chen, Zhongke Gu, Haiping Sun, Yuxuan Qi, Xuelin Qin, Jiansong Dai

**Affiliations:** 1 Sport Science Research Institute, Nanjing Sport Institute, Nanjing, Jiangsu, China; 2 Department of Sports and Health Science, Nanjing Sport Institute, Nanjing, Jiangsu, China; 3 Department of Fencing, Nanjing Sport Institute, Nanjing, Jiangsu, China; 4 School of Physical Education, Performance and Sport Leadership, Springfield College, Springfield, MA, United States of America

**Keywords:** Fencing, Lunge velocity, Squat jump, Countermovement jump, Drop jump, Isometric mid-thigh pull

## Abstract

**Objective:**

This study investigates the relationship between lower-body strength, power, and lunge velocity in elite female fencers of different weapons (foil, épée, and saber) to analyze the differences in fitness requirements between weapons and to provide a scientific basis for specialized fitness training.

**Methods:**

A cross-sectional study design was used to include 45 female fencers (12 in foil, 16 in saber, and 17 in épée) who performed the isometric mid-thigh pull test (IMTP), countermovement jump, squat jump, drop jump, static lunge velocity and advance lunge velocity tests. A one-way Analysis of Variance (ANOVA) was used to compare the differences among weapons groups, and Pearson’s correlation was used to analyze the relationship between lower-body strength, power, and lunge velocity.

**Results:**

Épée fencers had significantly higher advance lunge velocity than foil (*P* = 0.0003) and saber (*P* = 0.0001). The IMTP-50 ms rate of force development (RFD) of saber fencers was significantly better than that of épée fencers (*P* = 0.0213). Correlation analysis showed that the advance lunge velocity of foil fencers was strongly and positively correlated with IMTP relative maximal force (*r* = 0.63, *P* = 0.030). In contrast, the static lunge velocity of saber fencers was strongly correlated with squat jump height (*r* = 0.67, *P* = 0.004) and IMTP 250–300 ms RFD (*r* = 0.53, *P* = 0.035).

**Conclusion:**

Significant differences in lunge performance were found between fencers of different weapons. The results indicate that épée fencers exhibit significantly superior advance lunge velocity compared to foil and saber fencers. Furthermore, foil fencers require greater lower-body strength, and saber fencers rely more on the ability to develop force rapidly. The study supports the development of differentiated physical training programs for each weapon to optimize competitive performance.

## Introduction

Fencing is a combat sport dominated by technique, where fencers engage in rapid movement and varied tactical combinations on a 14-meter strip. The objective is to create optimal attacking opportunities and score points by precisely striking valid target areas with accurate sword movements. The sport features three weapons: foil, épée, and saber, each with its specific scoring rules. In épée, the entire body is valid for scoring through tip thrusts; foil limits targets to the torso, while saber allows slashing with both blade edge and tip, targeting upper body areas. These fundamental rule differences shape unique technical and physical demands across disciplines.

Lower-body strength, power, and lunge velocity represent fundamental athletic capacities in competitive fencing, influencing fencers ability to execute rapid displacements and precise attacks during bouts (Turner et al., 2014). While these physical attributes are recognized as essential determinants of fencing performance, research examining weapon-specific physiological demands remains limited. Although the International Fencing Federation (FIE) regulations stipulate identical bout durations across épée, foil, and saber disciplines, significant variations emerge in effective combat time, technical patterns, and physiological requirements due to differences in target areas and offensive strategies (International Fencing Federation, 2025). Empirical research indicates significant differences in effective combat time across fencing disciplines. Épée matches range from 53.9% to 62.5%, foil from 25.5% to 31.8%, and saber, with the lowest proportion, between 9.4% and 11.5% (Tarragó, Bottoms & Iglesias, 2023). Time-motion analyses of international competitions (Roi & Bianchedi, 2008; Aquili et al., 2013) revealed distinct work-to-rest ratios across disciplines: approximately 1–2:1 for épée, 1:3 for foil, and 1:6 for saber (Roi & Bianchedi, 2008; Aquili et al., 2013). The average attack duration per round further highlights these weapon-specific traits, with épée at 15 s, foil at 5 s, and saber at 2.5 s. Furthermore, analysis of competition-specific attack patterns revealed pronounced variations in frequency: women’s épée averages 11–28 attacks per bout, men’s épée 16–30, men’s foil 23–35, and saber averages a consistent 21 (Aquili et al., 2013). These differences, which reflect the unique competitive profile of each weapon, provide an essential theoretical foundation for subsequent studies on the athletic performance and physical fitness demands of fencers in each weapon discipline.

Most existing studies have focused on young fencers, while less attention has been paid to elite adult fencers. For example, Turner et al. examined young fencers’ lower-body power and lunge velocity, which represent fundamental fencers’ capacities in competitive fencing, influencing fencers’ ability to execute rapid displacements and precise attacks during bouts power and lunge performance (mean age 15.9 yr and 6.3 years of training). However, they did not find significant differences between weapons, which may be related to the lower age and training level of the participants in the study (Turner et al., 2017). In contrast, elite fencers have demonstrated a higher degree of specialization in neuromuscular coordination and reaction velocity, suggesting that the physical demands of different fencing disciplines may be more differentiated in high-level competitions. This characterization indicates that the physical demands of different sword types may be more differentiated at high levels of competition. In addition, although studies have examined the relationship between lower-body strength, power, and lunge performance, the findings are inconsistent, and there is still a lack of research, especially for elite fencers. This lack of research limits the scientific guidance of physical training for fencers in different weapons and affects the relevance and effectiveness of training programs.

This study aimed to analyze the lunge velocity, lower-body strength and power of elite female fencers, to explore whether there are significant differences between weapons, and to investigate the relationship between these physical fitness indicators and lunge velocity. By focusing on high-level fencers, this study reveals the specialized fitness needs of each weapon in competitive events. It provides a scientific basis for developing more targeted fitness training programs. This study also reveals the distinct physical demands associated with each weapon in competitive fencing and offers important references for coaches and researchers in related fields.

## Materials and Methods

### Participants

*A priori* power analysis was performed using G*Power 3.1 to determine the sample size required to detect between-group differences in the primary outcome of advance lunge velocity (ALV) *via* a one-way ANOVA. The calculation was based on an anticipated effect size of *f* = 0.21 (small-to-medium), an alpha level of 0.05, and a desired statistical power of 0.80, assuming homogeneity of variances (Turner et al., 2016). This analysis indicated a minimum total sample size of 34 participants. Forty-five elite female fencers from the China Fencing Academy, comprising members of the foil (*n* = 12; age 20.5 ± 2.97 years; height 170.61 ± 5.3 cm; weight 60.58 ± 7.39 kg; years of training 9.33 ± 1.56 years), saber (*n*  = 16; age 20.94 ± 3.3 years; height 174.25 ± 5.14 cm; weight 67.94 ± 7.53 kg; years of training 9.44 ± 2.48 years), and épée (*n* = 17; age 19.06 ± 3.11 years; height 175.18 ± 6.52 cm; weight 63.18 ± 9.24 kg; years of training 8.18 ± 2.32 years) teams were included in this investigation to ensure statistical robustness, there were no statistically significant differences in age (*P* = 0.1711), height (*P* = 0.1061), weight (*P* = 0.0622), or years of training (*P* = 0.2119) among the weapons. Inclusion criteria were as follows: (1) current registration as female competitive athletes with the Chinese Fencing Association; (2) documented top-eight placements in national championships within the preceding two competitive seasons (2023–2024); (3) absence of neuromuscular or orthopedic impairments affecting fencing performance for ≥180 days pre-assessment; (4) voluntary participation with signed informed consent. All test items were completed in one day. All participant screening and testing procedures were completed within a single testing period during April and May 2024. The experimental protocol received ethical approval from the Nanjing Sport Institute Human Research Ethics Committee (Ref: RT-2025-03).

### Procedures

This study was a cross-sectional study. Before testing, all subjects underwent a standardized warm-up of 10 min of moderate-intensity jogging and dynamic stretching. A computer-generated random assignment sequence was used to randomize the testing order of all subjects in this study, who were familiarized with each test maneuver 2–3 times and uniformly wore fencing training shoes during testing. All test items were completed in one day.

### Isometric mid-thigh pull

The isometric mid-thigh pull (IMTP) assessment was conducted using a calibrated force platform (Kistler 9260AA, Switzerland; 1000 Hz sampling rate) synchronized with manufacturer-specific MRAS software (v4.3) for data acquisition, and the data were low-pass filtered (Butterworth, second order, 10 Hz). Participants assumed standardized positioning with knee flexion angles maintained at 130°–140° (validated *via* digital goniometer), erect spinal alignment, and hands secured to an Olympic barbell using weightlifting straps to neutralize grip strength variability (Haff et al., 2015; Marcus et al., 2017). Following standardized auditory cues (“3, 2, 1, PULL!”), fencers executed three 5-second maximal effort trials separated by 2-minute passive recovery intervals. The average of the three trials was taken for analysis. The rate of force development (RFD) was calculated as the instantaneous slope of the force-time curve (RFD = ΔForce/ΔTime) over the 0–300 ms period following force onset (Banaszek et al., 2019). Absolute RFD (RFDabs) values were derived for the intervals of 0–50, 0–100, 0–150, 0–200, 0–250 and 0–300 ms. Peak force was defined as the maximum force recorded during the 5-second isometric test, with the participant’s body weight (in Newtons) subtracted (Waugh et al., 2014). Test–retest reliability (ICC) for IMTP relative peak force was 0.8196, indicating excellent reliability.

### Countermovement jump

Participants executed maximal-effort countermovement jumps (CMJ) on an instrumented force platform (Kistler 9260AA), adopting standardized initial positioning with feet shoulder-width apart and hands stabilized on the iliac crests (no arm swing was needed). The protocol mandated rapid eccentric descent to 90° knee flexion, followed immediately by explosive concentric propulsion without transitional pause. Three trials were administered with 2-minute passive recovery intervals, during which real-time visual feedback of vertical ground reaction forces (vGRF) was provided to ensure maximal effort. Peak power output normalized to body mass (W kg^−1^) was derived from the trial demonstrating the highest force production, consistent with established CMJ standardization protocols (Harty et al., 2020; Antonio Paoli et al., 2021). Peak power and peak force were normalized to body mass to eliminate the influence of body mass differences on the ability to generate force and power. The average of the three trials was taken for analysis. We selected the following metrics as our research variables: relative peak force, relative peak power, peak concentric velocity, jump height, ground contact time, and the modified reactive strength index (RSImod).

### Squat jump

From a static squat position (90° knee flexion measured by goniometer) with hands on the iliac crests (no arm swing was needed), subjects maintained the position for 3 s before executing a squat jump (SJ) without countermovement (Toro-Román et al., 2022). Three trials were conducted with 2-minute recovery periods. The mean value of the three successful trials was calculated. We selected relative peak force, relative peak power, jump height, and concentric peak velocity as the outcome measures. The average of the three trials was taken for analysis. Jump height was divided by time to take-off to calculate RSImod (McMahon, Jones & Comfort, 2022).

### Drop jump

A 40 cm plyometric box was positioned anterior to the force platform. The reliability of a 40 cm drop jump height has been established in research for measuring both the reactive strength index (CV = 3.0%, ICC = 0.95) and jump height (CV = 2.8%, ICC = 0.98)(Markwick et al., 2015). Subjects stood on the box with feet near the edge and hands on the iliac crests (No arm swing was needed). Upon initiation, one foot was extended beyond the box edge to initiate a controlled free-fall landing, followed by an immediate maximal vertical jump upon ground contact (Birat et al., 2020). Although a standardized 40 cm absolute height was used to ensure consistency across participants, all elite fencers were capable of performing this test safely. The drop jump (DJ) test was administered three times with 2-minute rest intervals between trials. The mean value of the three trials was calculated for subsequent analysis. We selected relative peak force, relative peak power, contact time, jump height, and RSImod as the study metrics. Jump height was divided by time to take-off to calculate RSImod (McMahon, Jones & Comfort, 2022). This study found that the drop jump height test demonstrated excellent test-retest reliability (ICC = 0.8695, CV = 0.19%).

### Lunge velocity

**Figure 1:**
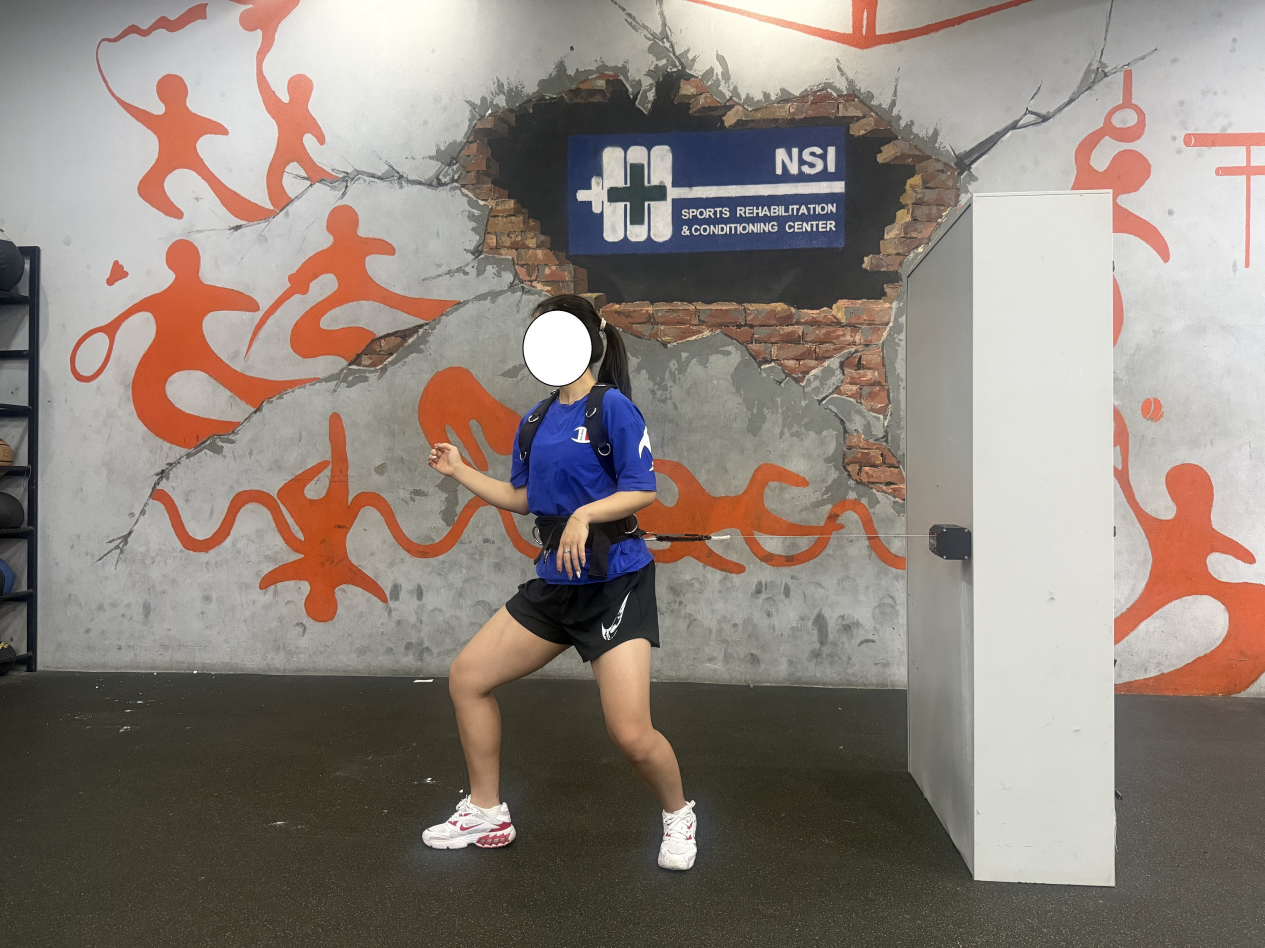
Schematic of the experimental setup for lunge velocity testing using the GymAware device.

Gymaware (Australia) is a velocity and power training test and evaluation system that effectively measures the velocity of movement for the exercise in question and is widely used in physical fitness training (Grgic et al., 2020; Orange et al., 2020; Wannouch et al., 2025). The Gymaware device has been used in research to measure and evaluate the velocity of the fencing lunge (Chen, 2020). The lunge velocity test was performed with the subject standing in the fencing en garde position. The Gymaware device was configured to measure linear velocity. The fixed end of the device was securely mounted on a vertical fixture positioned behind the athlete, aligned with the starting height of the athlete’s waist. The other end, with the retractable cord, was attached *via* a specialized belt to the side of the waist opposite the sword-holding hand, ensuring that the cord remained horizontal and aligned with the intended direction of the lunge movement. This setup allowed the device to measure the horizontal displacement velocity of the athlete’s center of mass during the lunge execution. Prior to data collection, the device was calibrated according to the manufacturer’s instructions, and a static test was conducted to verify a zero velocity reading in the en garde position (Fig. 1). The athlete did her best to complete the static lunge velocity (SLV) and advance lunge velocity (ALV) tests when ready, and each test was completed three times, with an interval of 2 min between trials, and the average of the three trials was taken for analysis.

The static lunge is a fundamental offensive technique initiated from an *en garde* position. This movement requires coordinated lower-limb kinetics: rapid plantar flexion driven by the rear leg combined with a swift anterior swing of the lead leg, generating anterior propulsion of the center of mass until lead-foot ground contact (Di Cagno et al., 2020). The advance lunge integrates forward displacement with static lunge execution. During displacement, the lead foot steps forward while the rear foot repositions, immediately transitioning into the static lunge action (Di Cagno et al., 2020).

### Statistical analyses

All outcome measures are presented as mean ± standard deviation (Mean ± SD). Normality and homoscedasticity assumptions were verified using the Shapiro–Wilk test (*α* = 0.05), with data confirming parametric assumptions (normality: *p* > 0.05; equal variances: *p* > 0.05). Between-group differences were analyzed *via* one-way ANOVA, followed by Tukey HSD (honestly significant difference) *post hoc* tests for pairwise comparisons. Bivariate relationships between lunge velocity, advance-lunge velocity, and performance variables were quantified using Pearson’s correlation coefficients (r), with effect magnitudes categorized as small (0.1 to 0.29), moderate (0.30 to 0.49), large (0.50 to 0.69), very large (0.70 to 0.89), near-perfect (0.90 to 0.99), and perfect (1.0) (Leary et al., 2012; Johansen et al., 2023). Statistical computations were performed using JMP Pro software (v17.0, SAS Institute). For multifactorial repeated measures, *p*-values were Bonferroni-corrected for multiple comparisons and evaluated against the standard significance threshold of *p* < 0.05.

## Results

Table 1 displays the mean values and standard deviations (SD) of static lunge velocity, advance lunge velocity, lower-body strength and power for foil, saber, and épée fencers. The weapon type had a statistically significant effect on advance lunge velocity (*F* = 16.7324, *P* < 0.001, *η*^2^=0.44345), CMJ contact time (*F* = 6.2786, *P* = 0.0041, *η*^2^=0.2301), and IMTP-50 ms RFD (*F* = 4.7785, *P* = 0.0135, *η*^2^=0.1853). *Post hoc* pairwise comparisons showed that épée fencers demonstrated significantly higher advance lunge velocity than foil (*P* = 0.0003) and saber (*P* = 0.0001). Saber fencers exhibited significantly greater CMJ contact time (*P* = 0.0035) and IMTP-50 ms RFD (*P* = 0.0213) than épée fencers.

**Table 1:** Lower-body strength, power, and lunge velocity of épée, foil, and saber fencers.

	Épée	Foil	Saber
Static Lunge Velocity (m s^−1^)	1.66 ± 0.14	1.60 ± 0.23	1.61 ± 0.17
Advance Lunge Velocity (m s^−1^)	2.85 ± 0.42^a^^b^	2.26 ± 0.31	2.17 ± 0.33
**Squat Jump**			
Relative Peak Force (N kg^−1^)	2.6 ± 0.19	2.52 ± 0.22	2.55 ± 0.28
Relative Peak Power (W kg^−1^)	56.59 ± 8.99	56.67 ± 8.88	57.31 ± 6.92
Jump Height (m)	0.31 ± 0.07	0.33 ± 0.06	0.3 ± 0.04
Concentric Peak Velocity (m s^−1^)	2.84 ± 0.41	2.85 ± 0.34	2.86 ± 0.18
**Drop Jump**			
Relative Peak Force (N kg^−1^)	2.71 ± 0.28	3.03 ± 0.55	3 ± 0.41
Relative Peak Power (W kg^−1^)	53.83 ± 4.67	53.76 ± 7.99	56.28 ± 4.84
Contact Time (s)	0.54 ± 0.1^a^	0.44 ± 0.08	0.46 ± 0.09
Jump Height (m)	0.35 ± 0.06	0.34 ± 0.06	0.36 ± 0.06
RSI_mod_ (m s^−1^)	1.19 ± 0.18	1.4 ± 0.28	1.4 ± 0.27
**Countermovement Jump**			
Relative Peak Force (N kg^−1^)	2.56 ± 0.19	2.44 ± 0.17	2.45 ± 0.2
Relative Peak Power (W kg^−1^)	50.12 ± 4.33	49.25 ± 4.86	50.19 ± 5.22
Concentric Peak Velocity (m s^−1^)	2.5 ± 0.22	2.5 ± 0.17	2.58 ± 0.18
Jump Height (m)	0.32 ± 0.05	0.33 ± 0.05	0.33 ± 0.05
Contact Time (s)	0.79 ± 0.1^b^	0.82 ± 0.1	0.91 ± 0.1
RSI_mod_ (m s^−1^)	0.73 ± 0.08	0.71 ± 0.1	0.68 ± 0.07
**Isometric Mid-Thigh Pull**			
Relative Peak Force (N kg^−1^)	2.21 ± 0.18	2.19 ± 0.14	2.19 ± 0.14
IMTP-50 ms RFD (N s^−1^)	2,449.51 ± 1,152.62^a^^b^	3,642.47 ± 1,248.13	3,704.45 ± 1,457.52
IMTP-100 ms RFD (N s^−1^)	2,571.79 ± 1,086.32	3,071.54 ± 850.00	3,302.49 ± 1,037.09
IMTP-150 ms RFD (N s^−1^)	2,539.34 ± 979.85	3,103.38 ± 817.16	2,931.38 ± 822.02
IMTP-200 ms RFD (N s^−1^)	2,432.68 ± 886.93	2,881.3 ± 595.39	2,676.12 ± 544.51
IMTP-250 ms RFD (N s^−1^)	2,666.17 ± 761.53	2,895.73 ± 491.07	2,951.89 ± 803.34
IMTP-300 ms RFD (N s^−1^)	2,867.21 ± 844.44	2,920.14 ± 579.13	3,052.13 ± 776.64

**Notes.**

a significantly different to foil (*p* < 0.05).

b significantly different to saber (*p* < 0.05).

IMTP isometric mid-thigh pull RFD rate of force development RSImod modified reactive strength index

**Figure 2:**
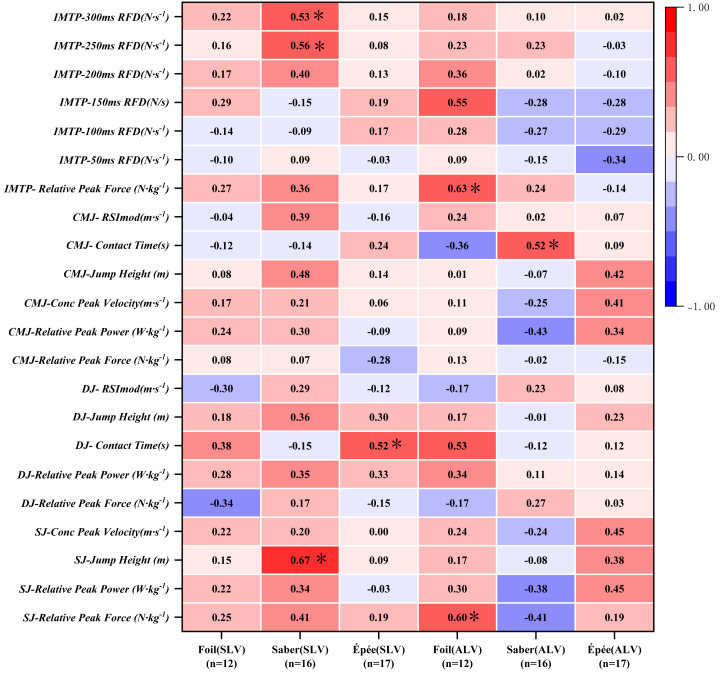
Pearson correlations between lunge velocity and lower-body strength/power metrics in fencers of different weapons.

Figure 2 reveals strong positive correlations in saber between static lunge velocity and SJ jump height (*r* = 0.67, 95% CI [0.2692–0.8771], *P* = 0.004), IMTP-250 ms RFD (*r* = 0.56, 95% CI [0.0928–0.8275], *P* = 0.023), and IMTP-300ms RFD (*r* = 0.53, 95% CI [0.0450–0.8118], *P* = 0.035). Advance lunge velocity in the saber correlated strongly with CMJ contact time (*r* = 0.52, 95% CI [0.0356–0.8086], *P* = 0.044). For foil fencers, advance lunge velocity showed strong positive correlations with SJ relative concentric peak force (*r* = 0.60, 95% CI [0.0427–0.8739], *P* = 0.038) and IMTP relative peak force (*r* = 0.63, 95% CI [0.0916–0.8850], *P* = 0.030), indicating lower-body strength significantly influences lunge performance in foil.

## Discussion

This study systematically compared elite female fencers lower-body strength and power characteristics across weapons, revealing weapon-specific factors influencing lunge velocity. Our data showed that épée fencers had significantly faster advance lunge velocity than foil (*P* = 0.0003) and saber (*P* = 0.0001). In contrast, saber fencers demonstrated a superior rate of force development (*P* = 0.0213). These findings diverge from Turner et al. (2017) “unified physical training” hypothesis, instead suggesting differentiated adaptation mechanisms aligned with competitive characteristics. Our data indicate that performance in foil may be more strongly associated with maximum lower body strength (IMTP relative peak force *r* = 0.63), while saber performance appears to be linked to the capacity for rapid force initiation (IMTP-RFD and static lunge velocity *r* = 0.52–0.67).

In foil, advance lunge velocity demonstrated strong positive correlations with squat jump relative peak force (*r* = 0.60, *P* = 0.038) and IMTP relative peak force (*r* = 0.63, *P* = 0.030), suggesting that lower-body strength may be a critical determinant of lunge performance in foil fencers. These findings align with the technical characteristics of foil, where the restricted valid target area (torso) necessitates precise movement control during rapid displacements, which relies fundamentally on robust lower-body strength. The IMTP test—a multi-joint isometric assessment widely used to evaluate lower limb strength and explosive power across sports disciplines—demonstrates good-to-excellent test-retest reliability for peak force measurements (Grgic et al., 2022). Numerous studies have identified strong correlations between IMTP force-time curve characteristics and lower limb strength, power, sprinting, change-of-direction ability, and sport-specific performance (Wang et al., 2016; Aben et al., 2020; Giles, Lutton & Martin, 2022). Additionally, the IMTP test has been applied to assess fatigue and post-competition recovery, as well as to evaluate physical traits in rugby union players and monitor seasonal variations in maximal strength (Darrall-Jones, Jones & Till, 2015; Aben et al., 2020; Hornsby et al., 2021). Given the strong associations observed in this study, IMTP relative peak force could be considered a key metric for assessing lunge capacity in foil fencers, particularly for tracking seasonal strength adaptations and fatigue recovery. Notably, no significant correlations between lunge velocity and explosive power metrics in foil suggest that maximal strength development may hold greater training priority than explosive power in this discipline.

The analysis of saber fencing demonstrated distinct performance characteristics. Static lunge velocity exhibited strong positive associations with squat jump height (*r* = 0.67, 95% CI [0.2692–0.8771], *P* = 0.004), IMTP-250 ms RFD (*r* = 0.56, 95% CI [0.0928–0.8275], *P* = 0.023), and IMTP-300 ms RFD (*r* = 0. 53, 95% CI [0.0450–0.8118], *P* = 0.035). Saber fencers displayed significantly greater IMTP-50 ms RFD than épée fencers (*P* = 0.0213), highlighting the importance of explosive force generation in saber fencing. These results correspond to the saber’s technical demands, where valid target zones (head, torso, upper limbs) and slashing techniques necessitate rapid movement initiation and efficient force application. Research confirms significant relationships between IMTP RFD and squat jump performance (*r* = 0.820, *P* < 0.05) (Haff et al., 1997), reflecting fencers neuromuscular capacity to produce high-force outputs rapidly. IMTP force-time metrics also correlate with sprint performance (Thomas et al., 2015; Townsend et al., 2019; Lum & Joseph, 2020): moderate associations with 20 m sprint velocity (RFD 0–200 ms: *r* = 0.485–0.493, *P* < 0.05) and strong negative correlations with 5 m/20 m sprint times (RFD 0–100 ms: r = −0.710 to −0.750; RFD 0–300 ms: r = −0.740 to −0.780, *P* < 0.01) (Thomas et al., 2015; Townsend et al., 2019). Similar patterns have emerged in golf, where IMTP RFD correlates with clubhead velocity (RFD 0–150 ms: *r* = 0.343; RFD 0–200 ms: *r* = 0.389, *P* < 0.05) (Wells et al., 2018), applied by Lum, Haff & Barbosa (2020) for monitoring strength adaptations in golfers. Given saber fencers’ superior IMTP-50 ms RFD and IMTP’s proven utility across sports, we recommend integrating IMTP testing into saber training regimens, prioritizing RFD0–50 ms as a key metric for assessing lunge performance and physical readiness.

The results of this study revealed differences in lunge velocity, lower-body strength, and power performance across weapons. Épée fencers demonstrated significantly higher advance lunge velocity compared to foil (*P* = 0.0003) and saber (*P* = 0.0001). Related studies have found that the offensive duration per bout in épée is 17.9 s, significantly longer than in saber (1.7 s) and foil (5.8 s), indicating that épée requires extended preparatory footwork before rapidly transitioning into lunge attacks (Aquili et al., 2013). The notably shorter CMJ contact time in épée fencers further emphasizes the importance of footwork transitions. These results suggest weapon-specific differences in the relationship between lower-body strength, power, and lunge velocity, which supports the development of tailored physical training programs to enhance performance in each weapon. There is disagreement on whether fencing training should be weapon-specific. It has been argued that the distinct demands of each weapon necessitate tailored physical training for optimal performance (Bottoms & Wylde, 2017). In contrast, other research has found no significant differences in relevant physical attributes among fencers from different disciplines, suggesting that specialized conditioning is not required (Turner et al., 2017). The discrepancies between our findings and Turner’s may stem from differences in testing metrics and athlete proficiency. Turner’s study used the RAL test to assess lower-body endurance (Turner et al., 2017). In contrast, this study focused on maximal single-effort lunge velocity as a measure of explosive power, reflecting distinct physical attributes. Additionally, our participants were elite fencers with longer years of training (8.9 years) whose neuromuscular systems had developed specialized force-production patterns, potentially enhancing explosive power in single-action attacks. In contrast, adolescent fencers (6.3 years of training) may lack such specialized adaptations (Turner et al., 2017). Research shows that elite fencers exhibit faster reaction times, shorter total response durations, and higher accuracy in lunge thrusts than novices (31), and more coordinated muscle synergies and movement patterns (Williams & Walmsley, 2000). Therefore, weapon-specific physical training likely holds greater practical value for elite fencers.

This study has several limitations that should be considered when interpreting the findings. First, regarding measurement methodology, although the Gymaware device has been used in fencing research and our testing setup was detailed, its specific configuration for assessing horizontal lunge velocity lacks direct validation against the gold standard of a 3D motion capture system. Second, the force-time data used to calculate metrics such as the rate of force development were sourced from the pre-processed output of commercial software. Future studies should collect and archive the raw force-time series signal to compute more reliable metrics, such as force at specific time epochs, enabling more robust analysis. Finally, as all participants were elite female Chinese fencers, the findings should be generalized cautiously to males, adolescents, or fencers from different training systems. Future research should employ more sophisticated measurement techniques, incorporate a broader range of kinetic metrics, and validate these findings across more diverse athletic populations.

The findings of our study provide targeted guidance for the training of elite fencers across weapons. For épée fencers, priority should be given to exercises that enhance rapid transition and explosive forward propulsion, such as drop jumps and integrated advance-lunge drills. For foil fencers, maximal lower-body strength is crucial; training should emphasize multi-joint lower-limb strength exercises including squats and deadlifts, alongside monitoring of IMTP peak force. For saber fencers, improving the rate of force development in the early phase is key, through high-velocity movements such as jump squats and power cleans, as well as IMTP-based training focused on RFD within 0–100 ms. These weapon-specific training approaches can help optimize the efficiency of fencing training and enhance athletic performance.

## Conclusion

This study reveals the specialized physical demands of elite female fencers across weapons, demonstrating significant differences in lunge velocity and lower-body strength profiles among foil, épée, and saber fencers. The results indicate that épée fencers exhibit significantly superior advance lunge velocity compared to foil and saber fencers, aligning with the discipline’s heightened requirements for attack distance and rapid advancement capacity. Furthermore, foil fencers show a strong correlation between lunge velocity and maximal lower-body strength, while saber fencers display close associations between lunge performance and rate of force development capability. These findings provide support for the development of weapon-specific conditioning programs that are tailored to the distinct biomechanical and neuromuscular characteristics associated with each weapon.

## Supplemental Information

10.7717/peerj.20879/supp-1 Supplemental Information 1 Primary data for the research paper

10.7717/peerj.20879/supp-2 Supplemental Information 2 The initial data used to calculate the ICC for the DJ and IMTP indicators ICC for calculating DJ and IMTP indicators

## Additional Information and Declarations

### Data Availability

The following information was supplied regarding data availability: The data are in the Supplementary File.
